# Ingestion of milk containing the Dp2 peptide, a dust mite allergen, protects mice from allergic airway inflammation and hyper-responsiveness

**DOI:** 10.1186/1710-1492-9-21

**Published:** 2013-06-13

**Authors:** Hsu-Chung Liu, Shun-Yuan Pai, Winston TK Cheng, Hsiao-Ling Chen, Tung-Chou Tsai, Shang-Hsun Yang, Chuan-Mu Chen

**Affiliations:** 1 Department of Life Sciences, Agricultural Biotechnology Center, National Chung Hsing University, Kuo Kuang Rd, Taichung, 402, Taiwan; 2 Division of Chest Medicine, Department of Internal Medicine, Chung Shan Medical University Hospital and School of Medicine, Chung Shan Medical University, Taichung, Taiwan; 3 Department of Animal Science and Biotechnology, Tunghai University, Taichung, Taiwan; 4 Department of Bioresources, Da-Yeh University, Changhwa, Taiwan; 5 Department of Physiology, National Cheng Kung University, Tainan, Taiwan; 6 iEGG Center, National Chung Hsing University, Taichung, Taiwan

**Keywords:** Transgenic mice, Allergen, Asthma, Immunotherapy, Group 2 allergen of *Dermatophagoides pteronyssinus*, Tolerance

## Abstract

**Background:**

Allergen-specific immunotherapy has been demonstrated to have potential for the treatment of allergic diseases. Transgenic animals are currently the best available bioreactors to produce recombinant proteins, which can be secreted in milk. It has not been clearly demonstrated whether milk from transgenic animals expressing recombinant allergens has immunomodulatory effects on allergic asthma.

**Methods:**

We aimed to determine whether the oral administration of milk containing a mite allergen can down-regulate allergen-specific airway inflammation. Transgenic CD-1 mice that express a recombinant group 2 allergen from *Dermatophagoides pteronyssinus* (Dp2) in their milk were generated using an embryonic gene-microinjection technique. Mouse pups were fed transgenic Dp2-containing milk or wild-type milk. Subsequently, these mice were sensitized and challenged with Dp2 to induce allergic airway inflammation.

**Results:**

Upon sensitization and challenge, mice fed transgenic Dp2 milk had decreased T-helper 2 (Th2) and increased T-helper 1 (Th1) responses in the airway compared with mice fed wild-type milk. Moreover, pre-treatment with transgenic Dp2 milk attenuated airway inflammation and decreased airway hyper-responsiveness.

**Conclusions:**

This study provides new evidence that oral administration of transgenic milk containing the Dp2 allergen down-regulated and moderately protected against allergic airway inflammation. Milk from transgenic animals expressing allergens may have potential use in the prevention of allergic asthma.

## Background

Allergic asthma is an inflammatory airway disease that occurs in response to allergen exposure. The disease is characterized by T-helper 2 (Th2) cell-dominated airway inflammation and airway hyper-responsiveness. The house dust mite is considered the most important indoor allergen affecting the development of asthma [1,2], and *Dermatophagoides pteronyssinus* is the predominant species of dust mite in Taiwan [3]. The 14-kD group 2 allergen isolated from *Dermatophagoides pteronyssinus* (Dp2) is considered a major allergen related to allergic asthma because the recombinant protein reacts with IgE in sera from 80% of mite-allergic patients [4].

Allergen-specific immunotherapy has been demonstrated to have therapeutic potential for the treatment of allergic asthma in many animal and clinical studies. The mechanism is related to a change in the immune response as a result of repeated allergen exposure. It has been demonstrated that immunotherapy induces T-helper 1 (Th1) cell differentiation in addition to down-regulating the Th2 cascade, and other studies have shown that regulatory T (Treg) cells play an important role in immunotherapy [5,6]. Subcutaneous injection immunotherapy (SCIT) has been shown to reduce the likelihood of developing asthma in both adults and children with rhinitis [7,8]. However, there are limiting factors associated with SCIT, such as anaphylactic reactions and the acceptability of injections [9]. Sublingual immunotherapy (SLIT), the administration of an allergen via the oral mucosa, has also been confirmed to reduce the incidence of new asthma cases [10]. The lower frequency of side effects and the relative convenience make SLIT a more acceptable treatment for children [11].

The human gastrointestinal tract is exposed to numerous dietary proteins, most of which are tolerated through suppression of the immune response in a process known as oral tolerance. Data from animal studies and early-phase clinical trials suggest that oral immunotherapy with an allergen is able to effectively induce tolerance and prevent food allergies [12]. To date, the effect of oral immunotherapy with allergens on the development of asthma has not been clearly identified.

Because the purification of Dp2 from dust mites is difficult, recombinant DNA techniques have been used to study allergen-specific immunotherapy [13,14]. Furthermore, our previous studies demonstrated that the mammary gland of transgenic mice can serve as a bioreactor to produce recombinant protein in the milk [15,16]. We therefore investigated transgenic mice expressing recombinant Dp2 in their milk. We hypothesized that the oral administration of transgenic Dp2-containing milk could induce tolerance and prevent allergic airway inflammation in a validated murine model of allergic asthma.

## Methods

### Construction of the αLA-CN-Dp2 transgene and production of transgenic mice

**Figure 1:**
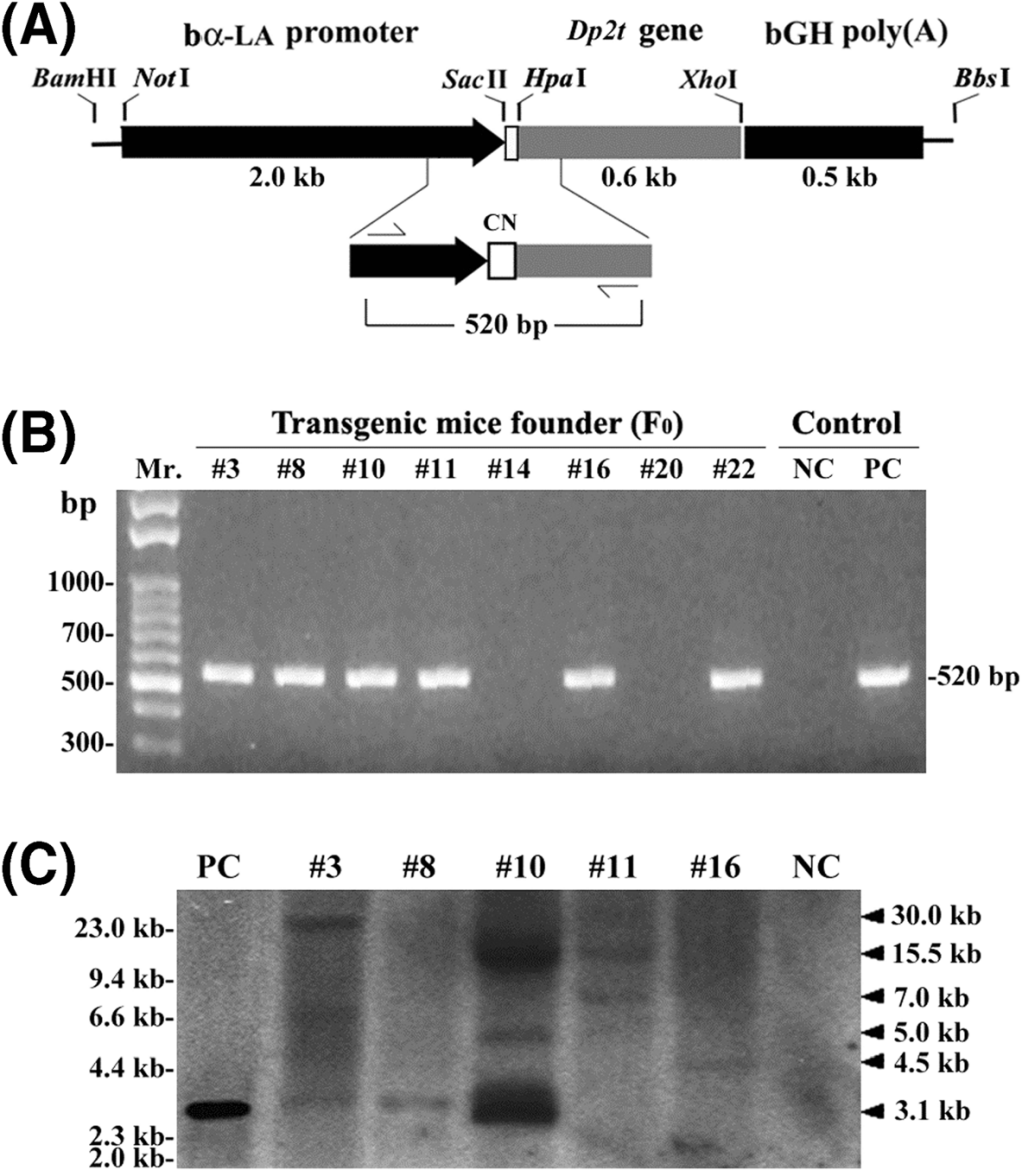
**Schematic map of the αLA-CN-Dp2t transgene construct and detection of its integration into transgenic mice.** (**A**) The structure of the αLA-CN-Dp2t-bGHpoly(A) fusion gene is shown. The construct includes the mammary gland-specific αLA promoter, the αS1-casein (CN) signal peptide leader sequence, the *Dermatophagoides pteronyssinus* mite truncated Dp2 (Dp2-t) cDNA, and the bovine growth hormone gene polyA signal. This construct was digested with *Bam*HI and *Bbs*I for microinjection. A primer pair for PCR screening was designed to flank the junction of the 3′-end of the αLA promoter and the 5′-end of the Dp2t cDNA. (**B**) Transgenic founder mice were identified through PCR screening. Mr. represents a DNA size marker of the 100-bp leader. (**C**) Southern blots showing the integration patterns of the transgene in the transgenic mice. PC represents pCR-αLA-CN-Dp2t plasmid DNA (positive control). NC represents normal mouse DNA (negative control).

The αLA-CN/pCR3 vector, which is a mouse mammary gland-specific expression vector, was used for transgene construction as previously described [15]. The 0.6 kb cloned Dp2 cDNA (GenBank accession number: AF276239) in the pGEM7 plasmid was used to generate a mature Dp2 coding sequence by PCR amplification using the primer set of Dp2-*Hpa*I(+) (5′-CGTTAACTCGTGATCAAGTCGAT-3′) and Dp2-*Xho*I(−) (5′-ACTCGAGGGTTTTCCCAGTCA-3′). The amplified products were double digested with *Hpa*I and *Xho*I (cutting sites are underlined in the above primer sequences) and then ligated into the αLA-CN/pCR3 vector. The 3.1 kb transgene fragment, consisting of the α-lactalbumin (αLA) promoter (2.0 kb), the αS1-casein (CN) signal peptide leader sequence (15-amino acids), the signal peptide-truncated Dp2 cDNA (Dp2t; 0.6 kb), and the bovine growth hormone (bGH) polyadenylation signal sequence (0.5 kb), was obtained from the αLA-CN-Dp2t/pCR3 vector with double digestion with *Bam*HI and *Bbs*I (Figure 1A). The transgene DNA was purified by CsCl2 gradient ultra-centrifugation, and transgenic CD-1 mice were generated by pronuclear microinjection as described previously [16,17]. The animal trials in this study were approved by the Institutional Animal Care and Use Committee of National Chung Hsing University, Taiwan (IACUC No.98-52).

### Detection of the transgene by PCR screening and Southern blot hybridization

Tail DNA from the founder mice was used to screen for the αLA-CN-Dp2t transgene by PCR amplification. PCR was performed using a set of primers, αLA-124(+) (5′-CTCTCTTGTCATCCTCTTCC-3′) and Dp2-395(−) (5′-CCAAAACACCATCATCACCC-3′), that amplified a 520 bp αLA-CN-Dp2t fusion gene. PCR was performed with 35 cycles of denaturation at 94°C for 30 sec, annealing at 55°C for 30 sec, and extension at 72°C for 45 sec in a thermal cycler (AG-9600; AcuGen systems, Lowell, MA). The PCR products were analyzed on a 1.5% agarose gel and detected by ultraviolet transillumination. The screening results were further confirmed by Southern blot hybridization as described previously [18]. Briefly, ten micrograms of genomic DNA was digested with StuI at 37°C overnight, electrophoresed on a 0.8% agarose gel, and transferred to a Durose nitrocellulose membrane (Stratagene, La Jolla, CA). The *Hpa*I-*Xho*I fragment of the Dp2 cDNA (0.6 kb) was used as a radioactive probe and hybridized to the membrane. Blots were subjected to autoradiography for one week at −20°C.

### Detection of recombinant Dp2 expression in the milk of transgenic mice

To identify the expression of recombinant Dp2 in the milk, the lactating female progeny of the transgenic mice were injected with oxytocin (China Chemical & Pharmaceutical Co., Ltd, Taipei, Taiwan), and milk was collected under anesthesia. Ten-fold dilutions of the milk samples collected during the different lactation stages were subjected to 12% SDS-polyacrylamide gel electrophoresis (SDS-PAGE) and stained with Coomassie blue. Milk proteins were electrotransferred from the gel to a PVDF membrane (MEN Life Science Products, Boston, MA). The expression of recombinant Dp2 was detected with a rabbit anti-Dp2 antibody (1:2000 dilution) and an anti-rabbit IgG second antibody conjugated with horseradish peroxidase (HRP) (1:10000 dilution). The blots were then visualized with the chemiluminescent ECLTM detection system (Amersham, UK) and exposed to X-ray film [18]. The levels of recombinant Dp2 in different lactating stages were quantified using an enzyme-linked immunosorbent assay (ELISA). A standard curve using purified Dp2 (Indoor Biotech. Ltd., UK) diluted from 5 mg/mL to 156.25 μg/mL was used to estimate the amount of recombinant Dp2 in the milk.

### Immunohistochemistry assay of mammary gland tissues

Freshly dissected mammary glands from transgenic and wild-type mice in the D15 lactating stage were fixed in paraformaldehyde as described [19]. Tissue sections (5 μm) were stained with hematoxylin and eosin (H&E) and photographed under a light microscope (Carl Zeiss, Germany). Immunohistochemical analysis was conducted as described [20,21]. Briefly, the Dp2 polyclonal antibody (LifeSpan BioSciences Inc., Seattle, WA, USA) was diluted 1:20 in 1% bovine serum albumin (BSA) in phosphate buffered saline (PBS), and 50 μl aliquots of anti-Dp2 were incubated with the tissue sections for 30 min at 37°C. After washing with 10 volumes of BSA-PBS buffer, the mammary gland slides were incubated with goat anti-rabbit IgG antibody conjugated with fluorescein isothiocyanate (FITC; Boehringer-Mannheim, Germany) at a dilution of 1:100 for 30 min at 37°C. After washing, the slides were observed under a Nikon Optiphot microscope (Nikon, Tokyo, Japan) equipped with epifluorescence optics.

### An animal model of allergic airway inflammation

The Dp2-containing milk was collected from different females of line #10 offspring, then pooled and quantified. The Dp2 concentration was adjusted to 2.0 mg/mL before feeding. Three-week-old CD-1 mice were obtained from the animal breeding center of the College of Medicine, National Taiwan University (the strain originated from the Jackson Laboratory, Bar Harbor, ME). These mice were divided into 3 experimental groups. (1) Group A: unsensitized mice fed wild-type (WT) milk (3.0 mL/kg body weight/day), which served as the normal control group. These mice were fed normal milk collected from WT mice and did not receive the subsequent sensitization with Dp2. (2) Group B: sensitized mice fed transgenic Dp2 milk (adjusted to 2.0 mg/mL Dp2 in the milk; 3.0 mL/kg body weight/day). These mice were fed milk collected from transgenic mice for 4 weeks and were then sensitized with an intraperitoneal injection of Dp2. (3) Group C: sensitized mice fed WT milk (3.0 mL/kg body weight/day). These mice were fed normal milk collected from wild-type mice and were then sensitized with Dp2. The sensitization was performed twice by intraperitoneal injection of 10 μg of purified Dp2 (Indoor Biotech. Ltd.) emulsified in 4 mg of Al(OH)3 on days 29 and 43. Al(OH)3 served as an adjuvant for induction of a good Th2 response. To induce allergic airway inflammation, the three groups of mice were challenged by exposure to an aerosol of 0.1% Dp2 for 30 min on day 50. All of the animal trails were repeated in two independent cohorts.

### Histopathological examination of lung alveoli tissues

After sacrificing the mice, the left lobes of the lung were dissected and inflated with 0.6 mL of 10% formalin for histological study. Paraffin sections prepared from the lungs were stained with hematoxylin and eosin for evaluation. We assessed the degree of alveolar congestion, hemorrhage, leukocyte infiltration, and the thickness of the alveolar wall [19].

### Analysis of pulmonary function

Eighteen hours after the aerosol challenge, mice were placed into a barometric plethysmograph (Buxco Electronics, Troy, NY) to measure their pulmonary function. Airway hyper-responsiveness was determined by measuring the Penh values (enhanced pause) when mice were exposed to an increasing dose of nebulized methacholine (Mch), as previously described [18,22]. The Penh values were calculated as the means ± SEM.

### Analysis of airway inflammation and cytokine expression

After the pulmonary function measurements were preformed, the experimental mice were sacrificed for the collection of bronchoalveolar lavage (BAL) fluid and lung sections. BAL fluid was collected using 500 μL of sterile endotoxin-free saline to wash the lungs. BAL cells were diluted in 1 mL PBS after centrifugation at 500 g at 4°C. The percentage of leukocytes among the BAL cells was determined with a hemocytometer. The cytospin preparation of 100 μL of BAL fluid was followed by staining with Liu stain [23] for total cell counts. The differential counts of BAL cells were performed under a microscope, and 200 total cells were counted. In addition, the supernatant of the BAL fluid was aspirated and stored at −80°C until the cytokine levels were assayed. The IL-4 and IFN-γ levels in the BAL fluid were determined using commercial ELISA kits according to the manufacturer’s instructions (BD Biosciences Pharmingen, San Diego, CA) [19].

### Determination of Dp2-specific IgE

Blood was collected from the tail vein of mice before sacrifice. The serum Dp2-specific IgE levels were determined using an ELISA kit. The plates were coated with 200 μL of purified Dp2 overnight at a concentration of 10 μg/mL in a 4°C refrigerator. After the sample serum was incubated with the plate for 2 h, a biotin-conjugated goat anti-mouse IgE detection antibody was added. The bound enzyme substrate was detected with streptavidin-alkaline phosphatase and p-nitrophenylphosphate (pNPP, Sigma, St. Louis, MO). After the chemical reaction, the optical density of the sample was measured at 405 nm in a multiscan spectrophotometer (BioRad, Hercules, CA).

### Statistical analysis

To assess the changes in the cell counts in the BAL fluid, in the cytokine contents, in the IgE levels, and in the level of airway hyper-responsiveness, repeated measures ANOVA was performed to compare the groups. Following ANOVA, the Duncan multiple range test was used to identify differences between the sensitized mice fed transgenic Dp2 milk and the sensitized mice fed WT milk. A value of *P* < 0.05 was used to indicate statistical significance.

## Results

### Generation of transgenic mice carrying the αLA-CN-Dp2 fusion gene

To generate transgenic mice capable of expressing recombinant Dp2 in their mammary glands, a DNA construct that carried a 2.0 kb regulatory sequence for the bovine-lactalbumin gene and the cDNA for a 0.6 kb mature Dp2 peptide with a leading αS1-CN signal sequence (15 amino acids) was engineered in the cloning vector pCR3 (Figure 1A). For pronuclear embryo microinjection, the mammary gland-specific cassette of the αLA-CN-Dp2t-bGH*polyA* construct was excised from the vector by double digestion with *Bam*HI and *Bbs*I. The microinjected embryos were transferred into the fallopian tubes of pseudopregnant mice. Six of the twenty-two newborn mice displayed a 520 bp PCR product indicative of positive transgenic mice (Figure 1B).

The number of integration sites in these transgenic mice was analyzed by Southern blotting using *Stu*I restriction enzyme digestion, which was used to cut the transgene-cellular junction. The results (Figure 1C) showed that the number of integration sites varied from one (Tg-#8 and Tg-#16) to three (Tg-#3 and Tg-#10).

### Successful expression of recombinant Dp2 in the milk of transgenic mice

**Figure 2:**
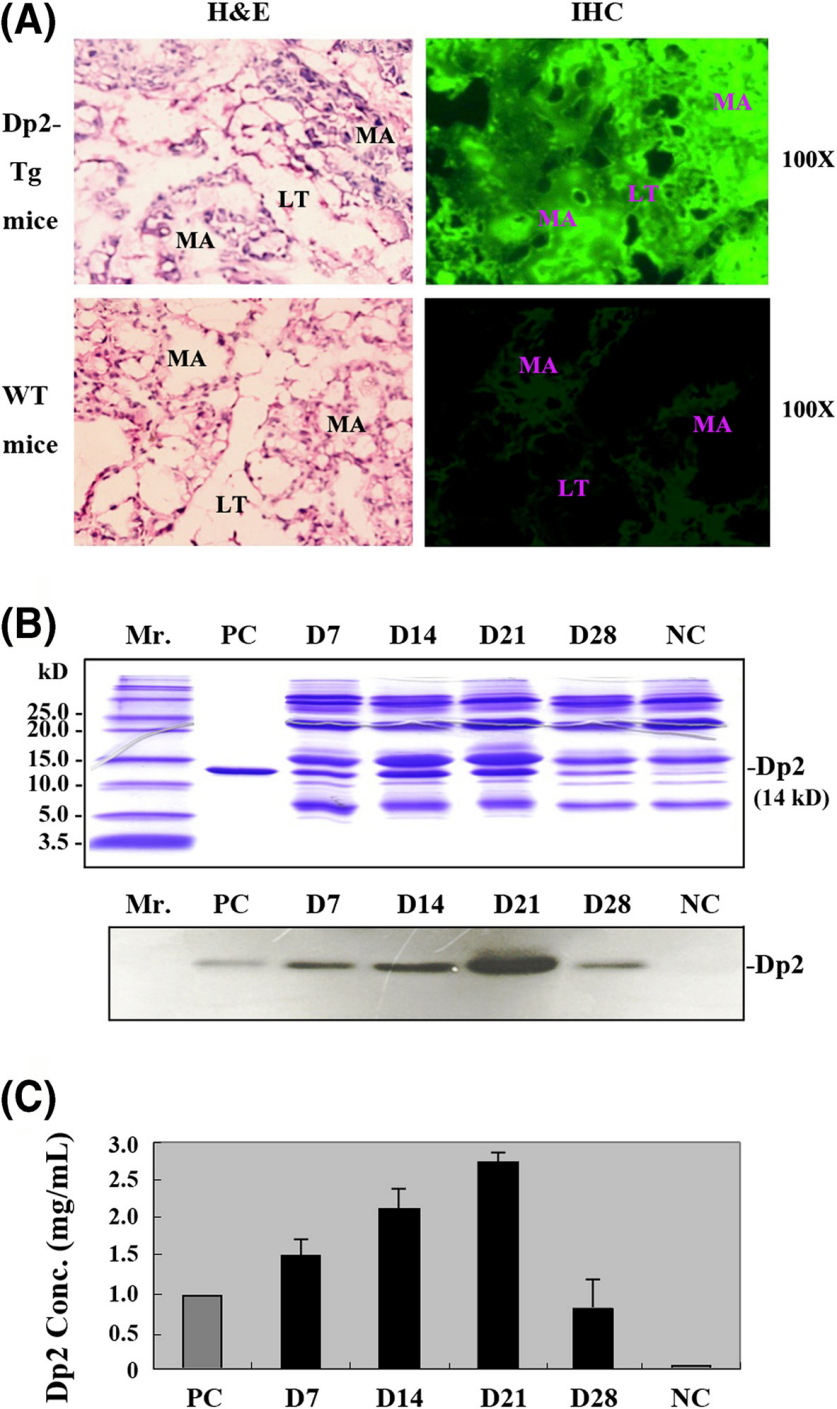
**Recombinant Dp2 expression in the transgenic mammary gland and secretion in milk.** (**A**) Freshly dissected mammary glands from Dp2-transgenic and wild type mice in the D15 lactating stage were fixed and stained with hematoxylin and eosin (H&E; left panel) and with antibodies for immunohistochemical (IHC; right panel) analysis. The structures of the mammary gland are labeled as ‘MA’ for mammary acini tissues and ‘LT’ for lactiferous tubules or secretary tubules. Samples were viewed using an epifluorescence microscope (original magnification, 100X). (**B**) Milk from transgenic female mice was collected, centrifuged, and subjected to SDS-PAGE (upper panel). The expression of the recombinant Dp2 protein was detected in this milk by Western blot analysis using a rabbit anti-Dp2 antibody (lower panel). D7 represents the transgenic milk that was collected from transgenic mice on 7th day of lactation (early stage). D14 and D21 represent the transgenic milk collected during the middle lactation stage. D28 represents the transgenic milk collected during the late lactation stage. Mr. represents a low-molecular-weight protein marker. PC represents the sample containing water and purified Dp2. NC represents the milk collected from wild-type mice. (**C**) Quantification of the amounts of recombinant Dp2 protein in the transgenic milk samples collected during different stages of lactation.

To analyze recombinant Dp2 expression in the transgenic mice, immunohistochemical (IHC) staining was performed on mammary gland sections. Data showed that recombinant Dp2 accumulated to high levels within the mammary acini and the lumen of the lactiferous tubules during the D15 lactating stage in Tg-#10 transgenic female offspring (Figure 2A, upper right panel). There were no detectable FITC signals or only a few green fluorescent background signals in the sections of mammary glands from lactating wild-type mice (Figure 2A, lower right panel). For further quantification of Dp2 secretion, milk was collected from lactating females of transgenic mice (Tg-#10) and wild-type control mice (NC) for SDS-PAGE (Figure 2B, upper panel) and Western blot (Figure 2B, lower panel) analyses. The assays were performed on milk samples collected in the early (Day 7), middle (Days 14 and 21), and late (Day 28) stages of lactation. The results showed that recombinant Dp2 was successfully expressed and secreted in the milk of the transgenic mice (Figure 2B). The transgenic milk collected during the middle lactation stage (Day 21) had the highest expression of recombinant Dp2 protein, with a concentration of 2.75 ± 0.21 mg/mL detected by ELISA (Figure 2C). Among four transgenic lines analyzed, the Tg-#10 line exhibited the highest Dp2 expression level. It contained three copies of the Dp2 transgene and stable germline transmission was demonstrated in offspring by slot-blot hybridization (Additional file 1). This transgenic mouse line was selected to breed offspring for further milk collection.

To characterize the tissue-specific expression of Dp2 mRNA transcripts, total tissue RNAs were extracted from different organs of transgenic lactating females. As shown in the Additional file 2, the Dp2 transcript, a 310 bp RT-PCR product, was found in the mammary gland (Ma) of lactating transgenic mice. No homologous transcripts were detectable in the brain (Br), heart (He), lung (Lu), liver (Li), spleen (Sp), kidney (Ki), ovary (Ov), muscle (Mu), or wild-type mouse mammary gland (NC-Ma). The results show that the Dp2 allergen was only expressed in the lactating mammary gland with no leaky expression in other tissues.

### Effect of transgenic Dp2 milk on airway hyper-responsiveness

**Figure 3:**
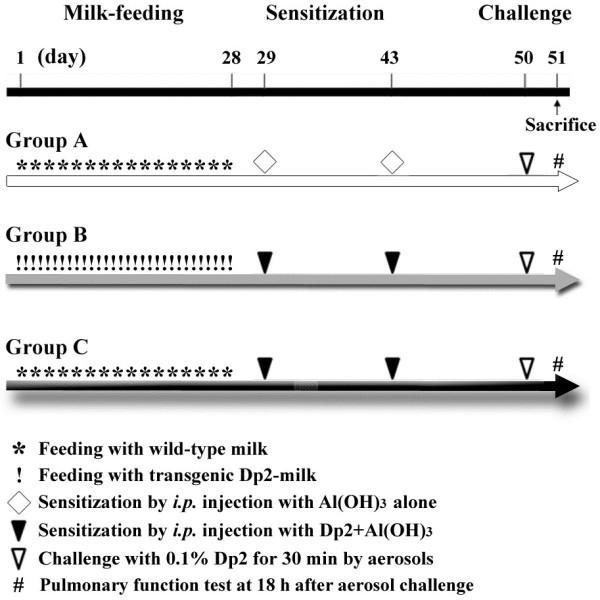
**Experimental protocol.** Transgenic Dp2 milk and wild-type (WT) milk were collected and fed to 3-week-old pups for 28 days. Mice were divided into three experimental groups. Two of the three groups of mice were sensitized by intraperitoneal injection of 10 μg of purified Dp2 on days 29 and 43. All mice were then challenged with 0.1% Dp2 aerosols on day 50. Mice underwent pulmonary function testing 18 h after aerosol challenge. BAL fluid, serum, and lung sections were collected on day 51 for analysis of the level of airway inflammation.

**Figure 4:**
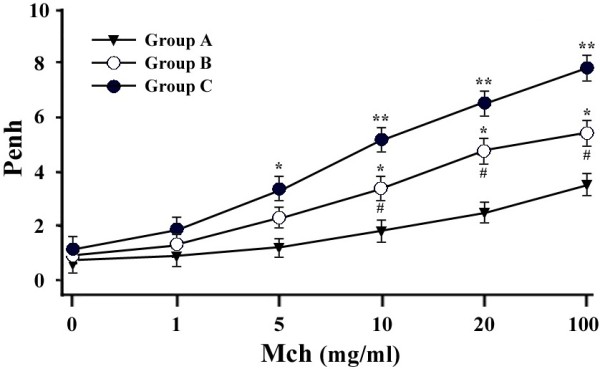
**The effect of transgenic Dp2 milk on airway hyper-responsiveness.** Individual mice were subjected to barometric whole-body plethysmography, and the enhanced pause (Penh) values were measured in response to challenge with different doses of nebulized methacholine (Mch). The data are presented as the means ± SEM. n = 5 mice per group with two independent experiments. * indicates *P* < 0.05 and ** indicates *P* < 0.01 for unsensitized mice compared with sensitized mice fed WT milk or Dp2 milk; # indicates *P* < 0.05 for sensitized mice fed transgenic Dp2 milk compared with sensitized mice fed WT milk.

The timeline of the animal trail to test whether the oral administration of transgenic animal milk containing the Dp2 allergen can down-regulate allergen-specific airway hyper-responsiveness and inflammation is shown in Figure 3. The levels of airway hyper-responsiveness in the three experimental groups were measured 18 h after Dp2 aerosol challenge. Sensitized mice fed WT milk (Group C) had higher Penh values than unsensitized mice (Group A) when mice were exposed to an increasing dose (10–100 mg/mL) of nebulized methacholine (*P* < 0.01). Thus, sensitized mice fed WT milk had increased airway hyper-responsiveness. In sensitized mice fed transgenic Dp2 milk (Group B), the Penh values were significantly reduced compared with the values in sensitized mice fed WT milk (*P* < 0.05; Figure 4). The results suggested that pre-treatment with transgenic Dp2 milk can reduce airway hyper-responsiveness in mice upon Dp2-induced allergic airway inflammation.

### Suppression of Dp2-induced airway inflammation by the ingestion of Dp2 milk

**Figure 5:**
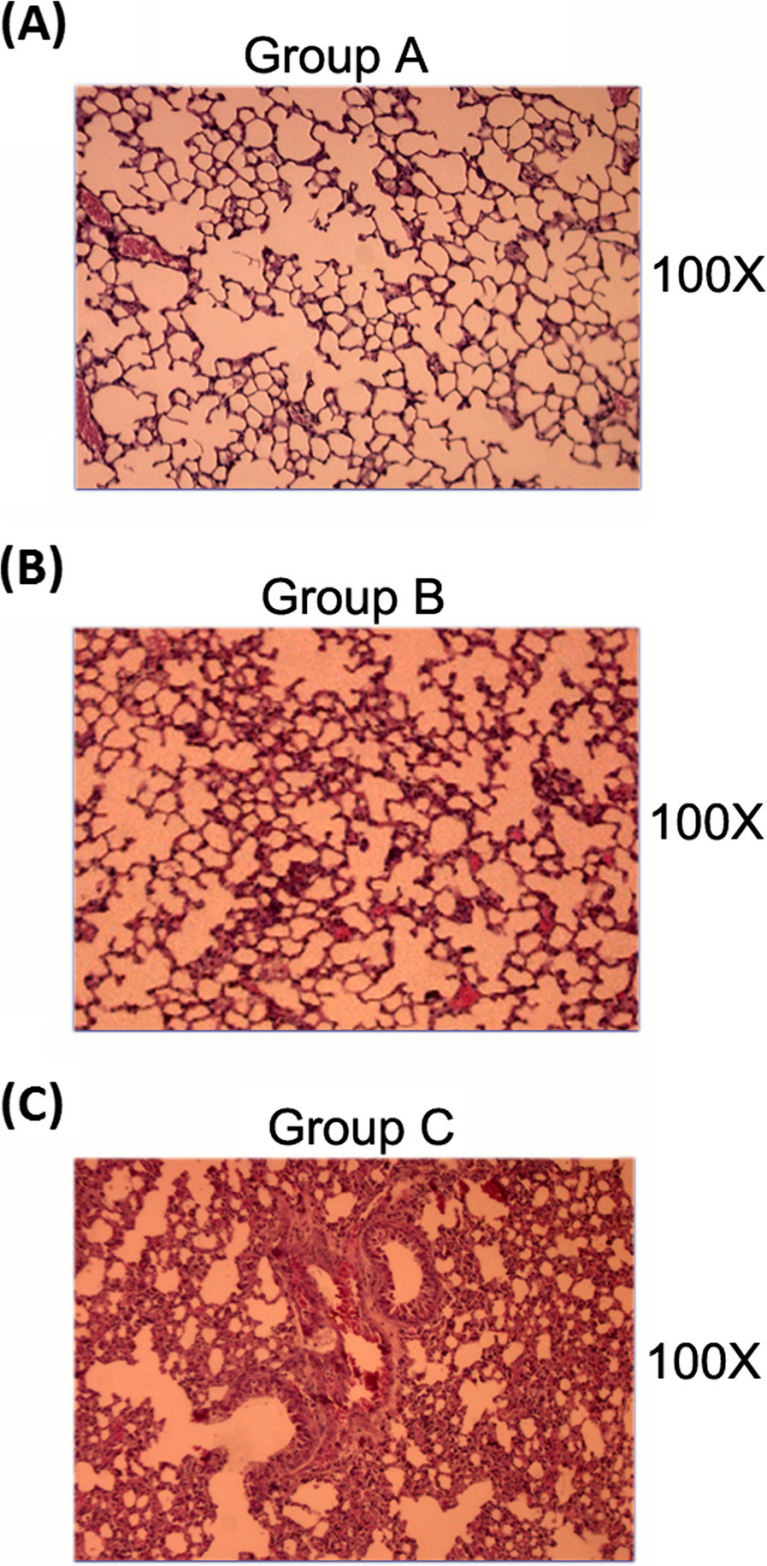
**Histopathological sections of lung tissues.** Lung alveoli with 100X magnification. (**A**) Group **A**: Unsensitized mice fed WT milk as the normal control group; (**B**) Group **B**: Sensitized mice fed transgenic Dp2 milk as the immunotherapy group; (**C**) Group **C**: Sensitized mice fed WT milk as the allergic airway inflammation group.

Inflammatory cell infiltration and epithelium damage in the lung alveoli tissues were examined by histochemical staining and compared among different experimental groups after Dp2 allergic challenge. We observed reductions in alveolar congestion, hemorrhage, leukocyte infiltration, and the thickness of the alveolar wall in the sensitized mice fed transgenic Dp2 milk (Group B; Figure 5B) compared with the sensitized mice fed WT milk (Group C; Figure 5C).

**Figure 6:**
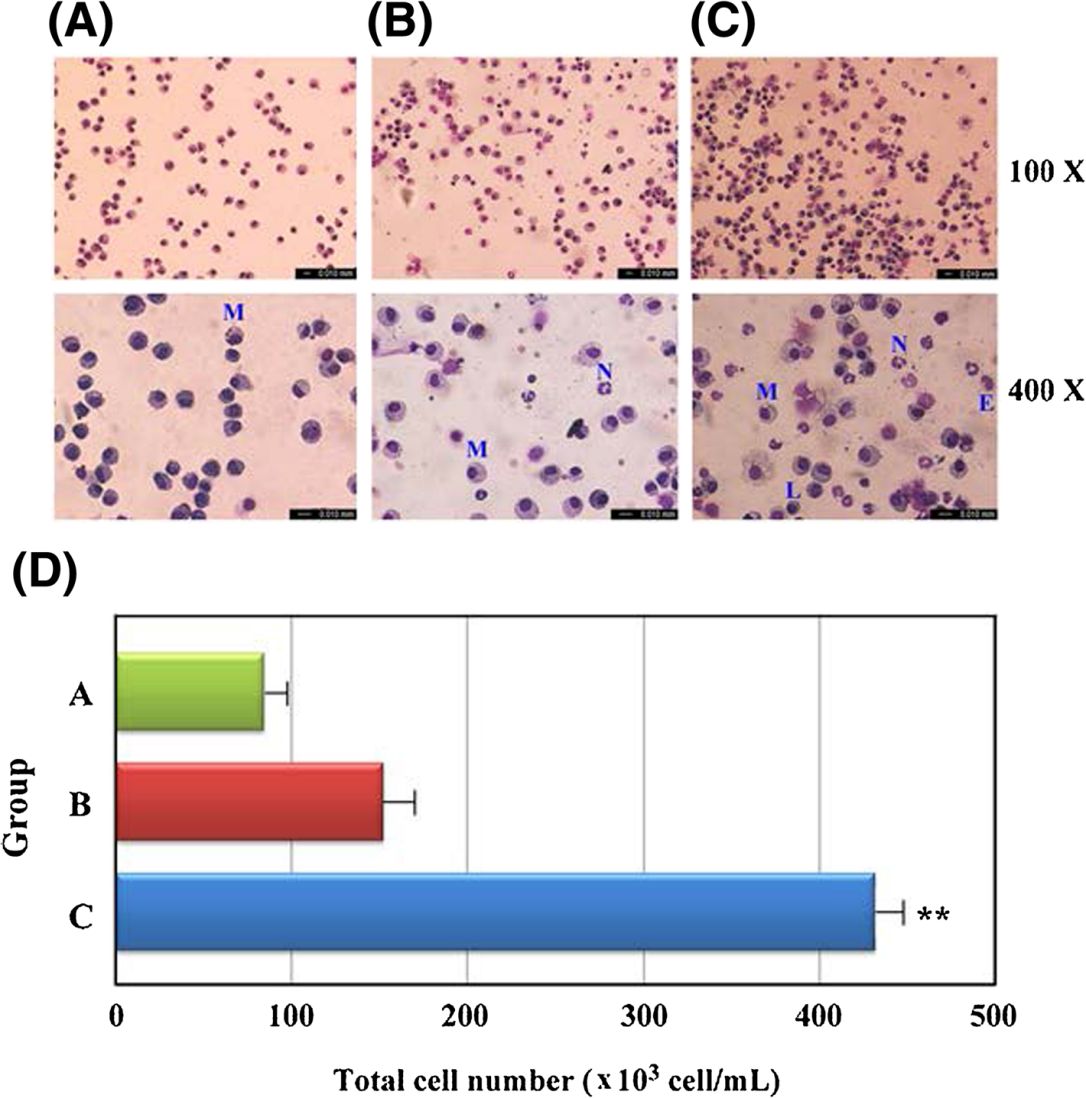
**Cytospin images of infiltrated cell types in the bronchoalveolar lavage (BAL) fluid of mice by Liu’s stain.** (**A**) Unsensitized mice fed WT milk as the normal control group. (**B**) Sensitized mice fed transgenic Dp2 milk as the immunotherapy group. (**C**) Sensitized mice fed WT milk as the allergic airway inflammation group. The images were observed under 100X (upper panels) or 400X (lower panels) magnifications. E: eosinophils; L: lymphocytes; M: macrophages; N: neutrophils. (**D**) Total infiltrated cell counts in BAL fluids from the three experimental gruops.

The leukocyte subpopulations in the BAL fluids were measured to evaluate the effect of pre-treatment with transgenic Dp2 milk. The results showed that sensitized mice fed transgenic Dp2 milk (Group B) had significantly fewer total lung infiltrated cells (*P* < 0.01), in their BAL fluid than sensitized mice fed WT milk (Group C), as demonstrated by the cytospin images in Figure 6A and the quantitative total infiltrated cell population counts in Figure 6B. In addition, significantly lower percentages of neutrophils (*P* < 0.05) and eosinophils (*P* < 0.05) were observed in the BAL fluids of Dp2 milk-treated mice compared with WT milk-treated mice (Additional file 3).

### Effect of transgenic Dp2 milk on bronchoalveolar lavage fluid cytokine levels

**Figure 7:**
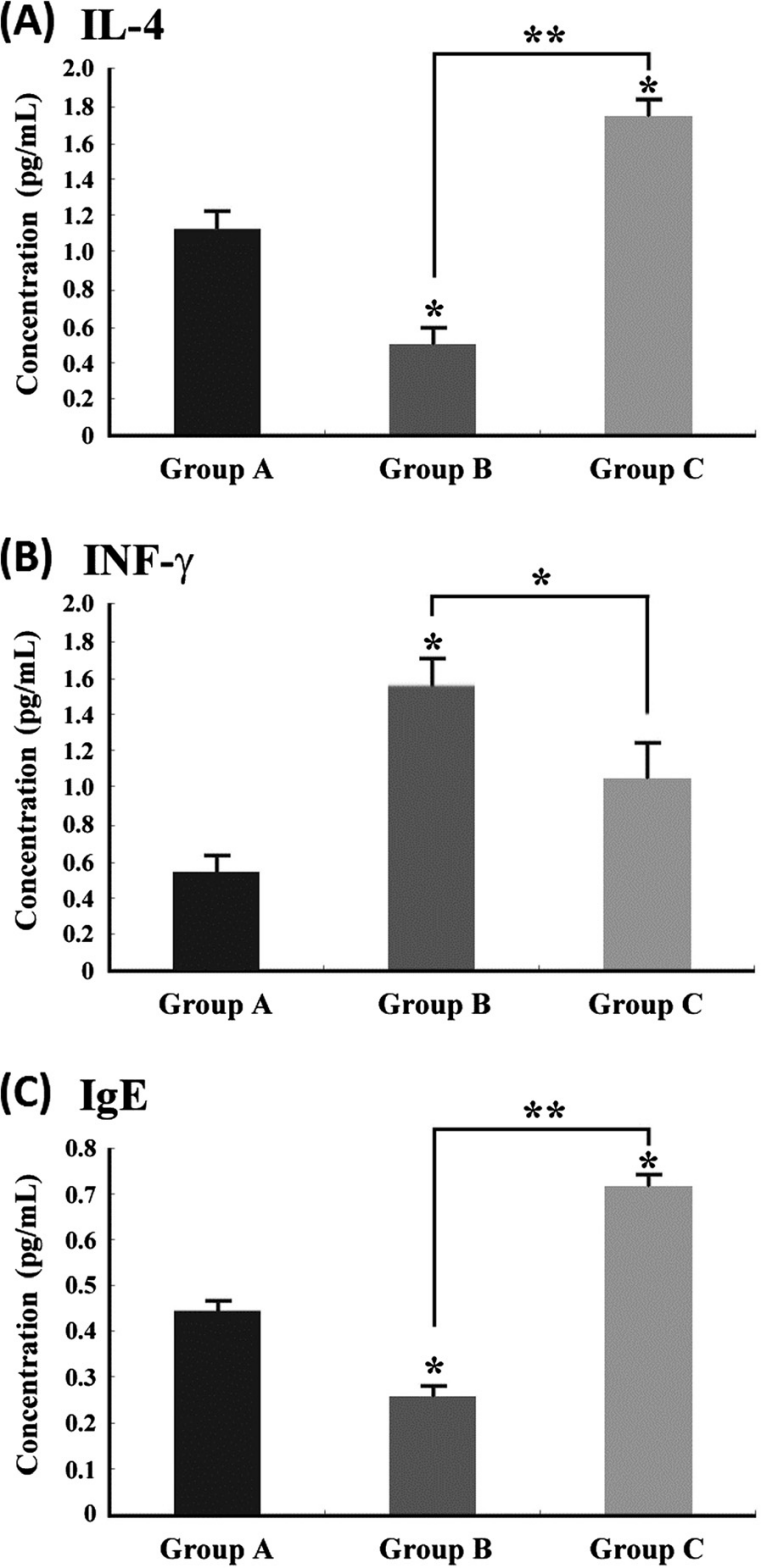
**Immunomodulatory effects of transgenic Dp2 milk on the expression of cytokines and allergen-specific immunoglobulins.** The supernatant of the BAL fluid from mice in the three experimental groups was analyzed by ELISA to determine the IL-4 (**A**) and IFN-γ (**B**) levels. Serum samples from mice in the three experimental groups were analyzed to determine the Dp2-specific IgE (**C**) by ELISA. The data are expressed as the means ± SD of values obtained for individual mice. n = 5 mice per group. (* *P* < 0.05; ** *P* < 0.01)

The levels of IL-4 (a Th2 cytokine) and INF-γ (a Th1 cytokine) were measured in the BAL fluid. Sensitized mice fed transgenic Dp2 milk (Group B) had significantly lower IL-4 levels than sensitized mice fed WT milk (0.537 ± 0.06 pg/mL vs. 1.734 ± 0.157 pg/mL; *P* < 0.01) (Figure 7A). We also observed a significant difference in IFN-γ levels between sensitized mice fed transgenic Dp2 milk and sensitized mice fed WT milk (1.572 ± 0.213 pg/mL vs. 1.046 ± 0.229 pg/mL; *P* < 0.05) (Figure 7B). These results demonstrate that pre-treatment with transgenic Dp2 milk not only suppressed the expression of Th2 cytokines but also attenuated the infiltration of inflammatory cells in the lungs of mice that were sensitized and challenged with the Dp2 dust mite allergen. Lung sections also showed attenuated inflammatory cell infiltration in both the lung parenchyma and the bronchus of sensitized mice fed recombinant Dp2 milk compared with sensitized mice fed WT milk (data not shown).

### Effect of transgenic milk on Dp2-specific IgE in the serum

The levels of Dp2-specific IgE in the sera of mice were measured by ELISA. The results showed that sensitized mice fed transgenic Dp2 milk (Group B, 0.257 ± 0.015 pg/mL) had lower serum IgE levels than sensitized mice fed WT milk (Group C, 0.717 ± 0.017 pg/mL; *P* < 0.01) and unsensitized normal mice (Group A, 0.443 ± 0.021 pg/mL; *P* < 0.05) (Figure 7C). These data demonstrate that mice given transgenic Dp2 milk had decreased IgE levels in the serum after sensitization and challenge with Dp2.

## Discussion

In this study, we engineered transgenic mice to express a recombinant Dp2 peptide and secrete it in their milk. The ingestion of this transgenic Dp2-containing milk attenuated Th2 responses in mice that were subsequently sensitized and challenged with purified Dp2 antigen. Both allergen-induced airway inflammation and airway hyper-responsiveness were decreased in sensitized mice fed transgenic Dp2 milk compared to sensitized mice fed WT milk. The immunomodulatory effect of transgenic Dp2 milk ingestion was associated with a decrease in IL-4 levels and an increase in IFN-γ levels. These findings suggest that the effect of pretreatment with transgenic Dp2 milk might be related to the down-regulation of the Th2 response and up-regulation of the Th1 response when these mice received a subsequent allergen sensitization and challenge. A limitation of our study is that the therapeutic intervention (the administration of transgenic Dp2 milk) occurred prior to intraperitoneal of the mice. It remains to be determined whether the ingestion of transgenic Dp2 milk could have an immunomodulatory effect on mice with established allergic airway inflammation.

Evidence from a previous animal study suggests that ovalbumin (OVA) antigen transfer through breast milk can induce immune tolerance and prevent allergic asthma [24]. The authors found that OVA antigen was efficiently transferred from the mother’s milk to neonatal mice, and the induction of tolerance relied on the presence of the allergen itself in the milk. This finding is in agreement with the immunomodulatory effect in our model of transgenic Dp2 milk ingestion. In our study, allergen expression in recombinant milk was demonstrated through IHC staining and Western blot analyses (Figure 2). The Dp2-containing milk produced by transgenic mice is convenient for breast feeding the newborns, but the milk uptake volume and Dp2 dosage are not easy to measure. Therefore, weaning age (three-week old) pups were used in this study so that we could carefully control the dose of orally ingested Dp2 doses. Furthermore, transgenic Dp2 milk was demonstrated to protect against allergic airway inflammation in this murine model of allergic asthma. In another study by Hsu et al. [25], Zucchini yellow mosaic virus (ZYMV) was used as a vector to express a dust mite Dp5 allergen in squash plants. The ingestion of an extract of this plant by mice previously sensitized to that allergen inhibited allergic airway inflammation and specific IgE synthesis. Our study was also based on the concept of producing a large amount of recombinant Dp2 allergen in a manageable genetically engineered system. However, in our study, the transgenic product was administered to mice before they underwent allergen sensitization, a protocol that differs from that used in the previous study [25]. The results of our study suggest that pre-treatment with transgenic Dp2 milk can down-regulate allergic airway inflammation. TGF-β and immunoglobulins in breast milk are thought to be important immunomodulatory factors affecting tolerance induction in neonates [24,26]. Further studies are needed to determine whether these immunomodulatory factors are important in the transgenic Dp2 milk.

The sensitized mice fed transgenic Dp2 milk still had inflammatory cell infiltration in the lung sections and increased airway hyper-responsiveness when compared with unsensitized normal mice. Therefore, the ingestion of transgenic Dp2 milk could induce a partial protective effect in mice. Mosconi et al. [27] showed that antigen-binding maternal IgG complexes in breast milk are more potent inducers of tolerance and prevention of asthma. The expression of TGF-β in breast milk has been demonstrated to be an important tolerogenic factor in tolerance induction [24,26]. It has been demonstrated that Th1 adjuvants, such as monophosphoryl lipid A and the immunostimulatory CpG motif in DNA, can enhance the effects of allergen-specific immunotherapies [28-30]. Further studies could attempt to enhance the protective effect of transgenic Dp2 milk by combining it with immunomodulatory factors or Th1 adjuvants.

The development of biotechnology that allows transgene expression in milk-producing animals has opened up new strategies to produce large amounts of a given recombinant protein in milk. The advantages of mammary glands as bioreactors are the higher production capability and the greater flexibility relative to other production systems [31,32]. Milk-secreted proteins are usually in the bio-active form as a result of proper posttranslational modifications, and these proteins can be easily purified for therapeutic use. Among the well-known recombinant proteins are human insulin-like growth factor 1 (IGF-1) [33], human growth hormone (GH) [34], recombinant tissue plasminogen activator (tPA) [35], human immunoglobulin [36], and human lactoferrin (hLF) [37]. Recombinant antithrombin (ATryn), the first pharmaceutical protein made from the milk of transgenic goats, has been approved by the European community and the U.S. Food and Drug Administration (FDA) for people with hereditary antithrombin deficiency [38]. The ingestion of transgenic Dp2 milk from dairy animals such as transgenic goats and cows would be a convenient and acceptable route of human administration. Our study demonstrated that the oral administration of transgenic Dp2 milk could be a feasible way to protect people from the development of allergic asthma.

## Conclusions

In summary, we have successfully produced transgenic mice expressing recombinant Dp2 peptide in their milk. Pre-treatment of mice with transgenic Dp2 milk can partially protect mice from allergen-induced airway inflammation and hyper-responsiveness. Our study may pave the way for designing new strategies to prevent allergic asthma in humans.

## Abbreviations

αLA: α-lactalbumin; ATryn: Antithrombin; BAL: Bronchoalveolar lavage; bGH: Bovine growth hormone; CN: αS1-casein; Dp2: Group 2 allergen from *Dermatophagoides pteronyssinus*; ELISA: Enzyme-linked immunosorbent assay; FITC: Fluorescein isothiocyanate; hLF: Human lactoferrin; HRP: Horseradish peroxidase; IGF-1: Insulin-like growth factor 1; IHC: Immunohistochemical staining; Mch: Methacholine; OVA: Ovalbumin; Penh: Enhanced pause value; SCIT: Subcutaneous injection immunotherapy; SLIT: Sublingual immunotherapy; tPA: Tissue plasminogen activator; ZYMV: Zucchini yellow mosaic virus.

## Competing interests

The authors declare that they have no competing interests.

## Authors’ contributions

Conceived and designed the experiments: WTKC, CMC. Performed the experiments: SYP, TCT, HCL. Analyzed the data: HLC, SHY. Wrote the paper: HCL, CMC. All authors contributed to data interpretation, and critically reviewed and approved the manuscript.

## Supplementary Material

Additional file 1 **Determination of Dp2 transgene copies in the genomes of transgenic (line Tg-#10) founder (F0) and its offspring (F1) by slot-blot hybridization.** (**A**) Ten micrograms of genomic DNA were blotted onto a nitrocellulose membrane and hybridized with a 0.6 kb Dp2 probe. (**B**) The filter was stripped and rehybridized with a 0.8 kb probe from the mouse β-actin gene that was used as an internal control. Copy standards (Std) were prepared by mixing 10 μg of non-transgenic tail DNA with a known amount of transgene plasmid DNA to produce transgene copy standards as shown in lane Std. Click here for file

Additional file 2 **Tissue-specific expression of the *Dp2 *transgene detected by RT-PCR.** Tissues were removed from lactating transgenic females at Day 14. Total RNA was isolated from mammary gland (Ma), brain (Br), heart (He), lung (Lu), liver (Li), spleen (Sp), kidney (Ki), ovary (Ov), muscle (Mu), and wild-type mouse mammary glands (NC-Ma). A non-lactation stage of mammary gland (dry) was used as a negative control. PC: positive control from αLA-CN-Dp2t plasmid DNA. The efficiency of DNase I treatment to eliminate DNA contamination was determined using total RNA from a transgenic mammary gland (tMa). When the reverse transcriptase was omitted from the reaction, no amplification was observed. A β-actin primer set was used as an internal control. Mr.: 100-bp ladder of DNA size marker. Click here for file

Additional file 3 **Differential cell counts in the bronchoalveolar lavage (BAL) fluid.** The percentages of eosinophils, neutrophils, lymphocytes, and macrophages in the BAL fluid were calaulated based on a total of 200 cells counted per slide using cytospin preparations stained with Liu’s stain. The data are presented as the means ± SD of values obtained from two independent experiments. n = 5 in each group. (* *P* <0.05). Click here for file
