# Supplementary figures and images for: Ingestion of milk containing the Dp2 peptide, a dust mite allergen, protects mice from allergic airway inflammation and hyper-responsiveness
Source: Allergy Asthma Clin Immunol. 2013 Jun 13;9(1):21. doi: 10.1186/1710-1492-9-21 (PMC3689609; doi:10.1186/1710-1492-9-21)

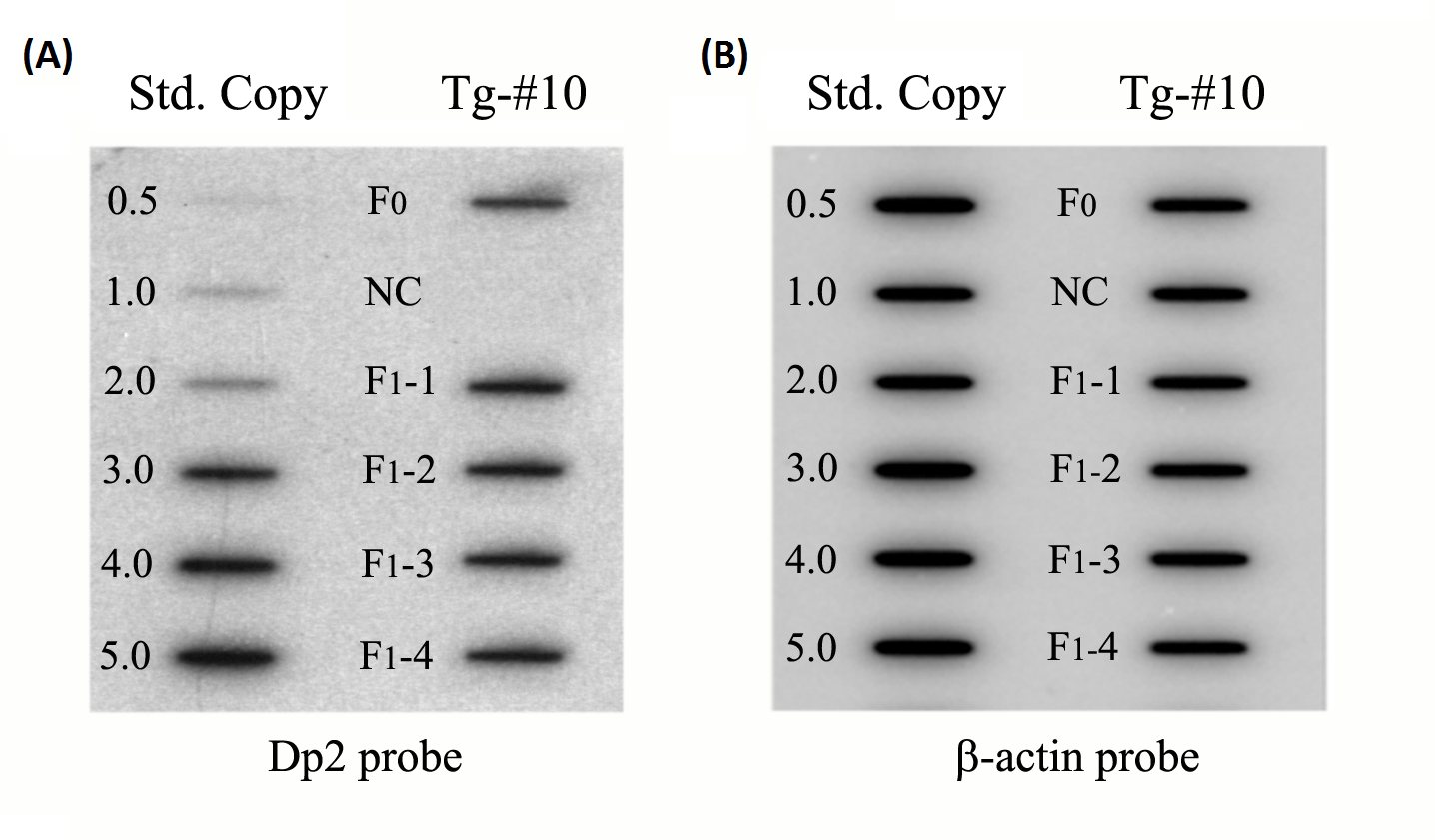

Supplement: Additional file 1 — Determination of Dp2 transgene copies in the genomes of transgenic (line Tg-#10) founder (F0) and its offspring (F1) by slot-blot hybridization. (A) Ten micrograms of genomic DNA were blotted onto a nitrocellulose membrane and hybridized with a 0.6 kb Dp2 probe. (B) The filter was stripped and rehybridized with a 0.8 kb probe from the mouse β-actin gene that was used as an internal control. Copy standards (Std) were prepared by mixing 10 μg of non-transgenic tail DNA with a known amount of transgene plasmid DNA to produce transgene copy standards as shown in lane Std. [file 1710-1492-9-21-S1.tiff]

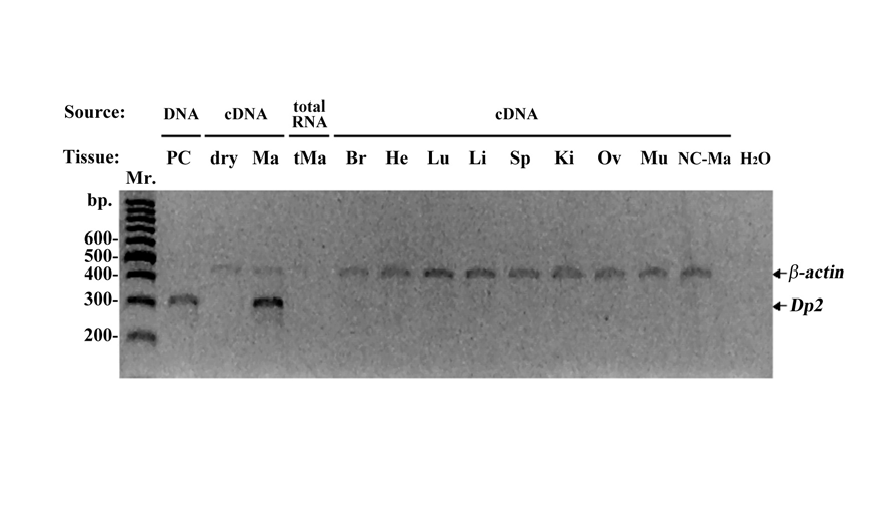

Supplement: Additional file 2 — Tissue-specific expression of the Dp2 transgene detected by RT-PCR. Tissues were removed from lactating transgenic females at Day 14. Total RNA was isolated from mammary gland (Ma), brain (Br), heart (He), lung (Lu), liver (Li), spleen (Sp), kidney (Ki), ovary (Ov), muscle (Mu), and wild-type mouse mammary glands (NC-Ma). A non-lactation stage of mammary gland (dry) was used as a negative control. PC: positive control from αLA-CN-Dp2t plasmid DNA. The efficiency of DNase I treatment to eliminate DNA contamination was determined using total RNA from a transgenic mammary gland (tMa). When the reverse transcriptase was omitted from the reaction, no amplification was observed. A β-actin primer set was used as an internal control. Mr.: 100-bp ladder of DNA size marker. [file 1710-1492-9-21-S2.tiff]

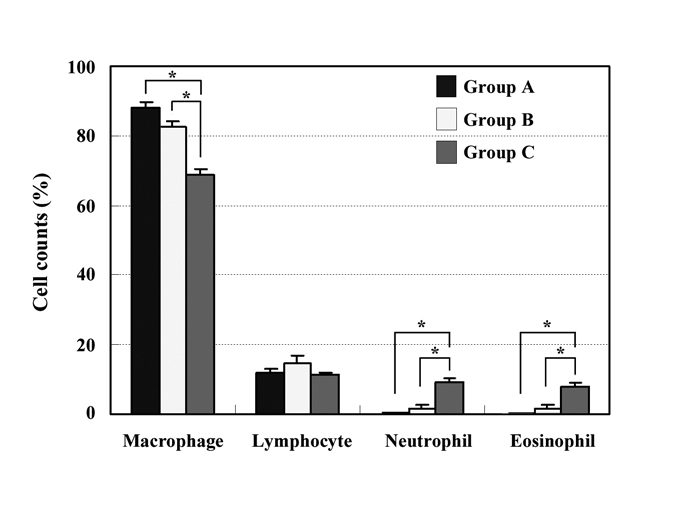

Supplement: Additional file 3 — Differential cell counts in the bronchoalveolar lavage (BAL) fluid. The percentages of eosinophils, neutrophils, lymphocytes, and macrophages in the BAL fluid were calaulated based on a total of 200 cells counted per slide using cytospin preparations stained with Liu’s stain. The data are presented as the means ± SD of values obtained from two independent experiments. n = 5 in each group. (* P <0.05). [file 1710-1492-9-21-S3.tiff]
